# Contaminant Exposure and Liver and Kidney Lesions in North American River Otters in the Indian River Lagoon, Florida

**DOI:** 10.3390/toxics12090684

**Published:** 2024-09-21

**Authors:** Ami Krasner, Megan Stolen, David Rotstein, Spencer Fire

**Affiliations:** 1 Department of Biological Sciences, Florida Institute of Technology, Melbourne, FL 32901, USA; sfire@fit.edu; 2 Blue World Research Institute, Cocoa, FL 32927, USA; 3 Marine Mammal Pathology Services, Olney, MD 20832, USA; drdrot@gmail.com

**Keywords:** microcystin, mercury, copper, trace elements, liver, kidney, Indian River Lagoon, sentinels, river otter

## Abstract

The harmful algal bloom (HAB) liver toxin microcystin (MC) and trace element biomagnification were previously detected in organisms in the Indian River Lagoon (IRL), Florida. Since there are no routine screening programs for these contaminants, liver tissue from North American river otters (*Lontra canadensis*), an important sentinel species in the IRL, was screened for MC via enzyme-linked immunoassay (ELISA), followed by confirmatory analyses via liquid-chromatography/mass spectrometry methods (LC-MS/MS). Liver and kidney samples were evaluated for trace element (As, Cd, Co, Cu, Fe, Hg, Mn, Mo, Pb, Se, Tl, and Zn) bioaccumulation via inductively coupled plasma mass spectrometry (ICP-MS). Histopathologic evaluation of the liver and kidney was conducted to assess possible correlation with toxic insults. Forty-three river otters were evaluated (2016–2022). Microcystin was not detected in any river otter sample (*n* = 37). Of those tested for trace element bioaccumulation (*n* = 22), no sample measured above provided reference ranges or estimated toxic thresholds for this species. There were no statistically significant patterns observed based on season, year, or age class, but sex had a small influence on trace element levels in the kidney. One individual had a kidney Cu level (52 μg/g dry weight) higher than any previously reported for this species. Trace elements were detected at presumed background levels providing baselines for future monitoring. For otters with available histopathologic evaluation (*n* = 28), anomalies indicative of contaminant exposure (non-specific inflammation, necrosis, and/or lipidosis) were present in the liver and kidney of 18% and 4% of individuals, respectively. However, since these lesions were not linked to abnormal trace element bioaccumulation or MC exposure, other causes (e.g., infectious disease) should be considered.

## 1. Introduction 

Natural and anthropogenic contaminants are escalating threats to the health of humans and wildlife that inhabit estuaries worldwide [1]. These include numerous harmful algal bloom (HAB) toxins and trace elements. Estuarine megafauna can serve as barometers of contaminant exposure and potential impacts on other animals, public health, and ecosystem health [1,2]. 

The Indian River Lagoon (IRL) watershed is a key geographic region where exposure to both HAB toxins and trace elements is an emerging concern for human and wildlife health [2,3]. This diverse, urban, estuarine ecosystem on the Atlantic coast of central Florida provides critical habitat to over 4000 species of flora and fauna, including commercially important fish and wildlife of conservation priority [2,3,4,5,6,7,8]. A primary threat to IRL health over the last several decades is the degradation of water quality due to limited contaminant flushing in conjunction with urban expansion, industrial and agricultural inputs, freshwater discharges, and stormwater, wastewater, and lawn waste run-off [9,10]. While these processes introduce numerous contaminants of concern into the IRL, including perfluoroalkyl substances (PFAS), herbicides, pesticides, microplastics, thermal pollution, medications (e.g., caffeine), and fecal coliform bacteria, key drivers of impairment are trace element and nutrient (i.e., nitrogen and phosphorous) pollution, the latter of which can contribute to numerous HAB events annually [9,10]. The health impacts of contaminant exposure to IRL inhabitants are often difficult to ascertain [3]. Yet, bioaccumulation of multiple HAB toxins and trace elements has been detected in IRL megafauna and is associated with disease, stranding events, and mortality [3,8,11,12,13,14,15,16,17,18,19,20,21]. 

Microcystin (MC), a liver toxin and tumor-promotor produced by cyanobacteria, was introduced to the southern IRL by freshwater outflows from Lake Okeechobee in 2005 [3,22,23,24]. Since then, MC has been detected in southern IRL water samples [23,25,26,27,28], human nasal swabs [28], putatively healthy, free-ranging bull sharks (*Carcharhinus leucas*) [3], and stranded green sea turtles (*Chelonia mydas*) from the lagoon [8]. Microcystin was also associated with an increased incidence of non-alcoholic liver disease among IRL human residents [29]. In 2018, six domestic dogs (*Canis lupus familiaris*) died from acute liver failure caused by MC contamination in the southern IRL [24]. Lethal exposure from MC has been documented in a key marine mammal species (southern sea otter, *Enhydra lutris nereis*) in central California [30], suggesting that such exposure may also be of concern for aquatic mammals in the IRL. However, there are no reports to date of MC exposure or related health impacts in aquatic mammals inhabiting the lagoon.

While many trace elements, including cobalt (Co), copper (Cu), iron (Fe), manganese (Mn), molybdenum (Mo), selenium (Se), and zinc (Zn), are essential to healthy bodily function, toxicity can occur when these elements are present at abnormal levels [31]. Toxicity can also occur when those that are non-essential, such as arsenic (As), cadmium (Cd), lead (Pb), mercury (Hg), and thallium (Tl), bioaccumulate [31]. Trace element pollutants of particular concern to IRL health include Cu and Hg. Anti-fouling paints used to protect ship hulls are likely responsible for increased Cu concentrations in IRL water samples and invertebrates [32,33]. Florida manatees (*Trichechus manatus*) in the IRL demonstrate higher Cu levels compared to those in other regions [16]. Hg biomagnification in IRL prey species and marine mammals has been linked to liver anomalies in common bottlenose dolphins (*Tursiops truncatus truncatus*), as well as Hg bioaccumulation in human hair samples that exceeded U.S. EPA exposure thresholds [17,18,20,21,34,35]. Sources of Hg to the IRL may include stormwater run-off, fossil fuel combustion, and municipal discharges [33]. Other persisting or escalating trace element contaminants in the system include As, Cd, Pb, and Zn [32,33].

Since IRL residents, domestic animals, and wildlife are not routinely screened for MC or trace element bioaccumulation, this study aimed to determine if a sentinel species, North American river otters (*Lontra canadensis*), had detectable (1) MC exposure, (2) trace element bioaccumulation, or (3) liver and kidney lesions suggestive of toxicity. No information was available (to the author’s knowledge) on trace element pollutants or HAB toxin exposure in the IRL river otter population. As accessible apex predators with sensitivity to many environmental contaminants, river otters are excellent candidates for such biomonitoring [36,37]. Study findings reported herein may serve as baselines for future monitoring and inform conservation efforts to better protect public welfare and wildlife inhabitants of this estuary of national significance.

## 2. Materials and Methods

### 2.1. Study Area and Animals

Just over 250 km long (902 km^2^) and ranging in width from several meters to nearly 9 km, the IRL covers almost half of Florida’s eastern coastline with a watershed that lies within seven counties [23,38]. The IRL primarily encompasses three estuaries: the Banana River, Indian River, and Mosquito Lagoon [38]. The lagoon’s southern boundary is Jupiter Inlet, while the northern boundary (Ponce de Leon Inlet) has a planned extension to include the Halifax River up to High Bridge Rd in Volusia County [9]. Deceased river otters found as road victims within the IRL watershed and reported to the Otter Spotter Program were considered for inclusion in this study. Additionally, one individual was included who was observed roadside, transferred to a local wildlife rehabilitation center for treatment, and died naturally after 128 days in care. Sex was determined through an examination of internal reproductive tissues [39]. Age class determination in river otters is most accurate when based on analysis of tooth cementum [40]. As these data were incomplete for our study population, age class estimations were performed based on total body length: juveniles ≤ 90 cm and adults > 90 cm [41]. Season of river otter carcass recovery was classified as follows: winter = December–February, spring = March–May, summer = June–August, and fall = September–November [42]. 

### 2.2. Sample Collection

Liver and kidney samples were collected during a routine necropsy. All available liver and kidney samples were tested. For contaminant analysis, approximately 1–30 g of tissue was collected utilizing established protocols [43]. Samples were stored in Whirl-pack bags and frozen at −20 °C until transfer and analysis. For histopathology, samples of liver and kidney from code 2 to 3 animals were placed in 10% formalin, processed routinely, sectioned at 5 to 7 μm, and stained with hematoxylin and eosin per established protocols [43]. Specimen condition was based on established methods: code 2 (fresh dead) and code 3 (moderate decomposition, organs intact) [43]. Sample collection, storage, and transfers were performed under established permits by the Otter Spotter Program (Cocoa, FL, USA), Florida Institute of Technology (FIT, Melbourne, FL, USA), GreenWater Laboratories (Palatka, FL, USA), Michigan State University (MSU, Lansing, MI, USA), and Marine Mammal Pathology Services (Olney, MD, USA).

### 2.3. Microcystin Extraction and Analysis

Microcystin screening was performed at FIT via the MCs/NODs ADDA-ELISA (Abraxis©; PN 520011; Warminster, PA, USA). The original sample was extracted. Liver was extracted by combining 1 g homogenized sample with 4 mL of extraction/elution solvent (90% MeOH/10% acidified water; acidified water = 99.9% deionized (DI) water/0.1% trifluoroacetic acid (TFA)) into a 15 mL polypropylene centrifuge tube. This extract was vortexed for 30 s and then centrifuged at 3400× *g* for 10 min. The extract was then diluted and precipitated by combining 1 mL of the extract supernatant and 2 mL of solid-phase extraction (SPE) diluent (99.85% DI water/0.1% formic acid/0.05% TFA) in a 20 mL glass culture tube, which was then vortexed. If the combined solution turned cloudy, it was centrifuged again at 3400× *g* for 5 min. The extract then underwent SPE cleaning as follows: (1) a 10 mL C18 500 mg solid-phase SPE column was conditioned by passing 10 mL of MeOH, followed by 10 mL of acidified water, through the column under vacuum pressure; (2) the 3 mL of diluted extract was passed through the SPE column under vacuum pressure; and (3) the SPE cleaned extract was eluted by passing 2 mL of the extraction/elution solvent through the column under vacuum pressure. 

Extracts were frozen at −20 °C in a 5 mL glass vial until ELISA analysis on a 96-well plate following manufacturer instructions. In addition to the six standards (0, 0.15, 0.4, 1, 2, and 5 ng/mL) and control (0.75 ± 0.185 ng/mL) included with the ELISA kit, three additional standards (0.015, 0.05, and 3.5 ng/mL) were prepared by diluting standard 1 or 5 as appropriate with MC diluent buffer. Liver samples were analyzed at a 1:10 dilution using MC sample diluent. Dilutions were chosen to minimize false negatives as this immunoassay was for screening purposes. Standards, controls, and sample extracts were run in duplicate. Absorbances were read at 450 nm using a microplate ELISA spectrophotometer. In general, the minimum detection limit (MDL) was 7 ng/g.

Selection criteria for confirmatory testing with the methyl-3-methoxy-4-phenylbutyric acid technique (MMPB) included those samples that (1) tested positive via ELISA and/or (2) had liver lesions potentially consistent with MC toxicosis (see below). Confirmatory testing was performed at GreenWater Laboratories. The oxidation and analysis for total MCs and nodularins was implemented as described in [24,44]. 

### 2.4. Trace Element Analysis

The concentrations of twelve trace elements (As, Cd, Co, Cu, Fe, Hg, Mn, Mo, Pb, Se, Tl, and Zn) in liver and kidney samples were measured at MSU Veterinary Diagnostic Laboratory. All samples were standardized by dry weight (dw) fraction calculated by gravimetry. Trace element contents in specimen tissues were determined via inductively coupled plasma mass spectrometry (ICP-MS). 

### 2.5. Health Analysis

Findings from gross and microscopic liver and kidney histopathology reports were included in this study when available to assess potential health impacts linked with MC exposure (defined as MC detection in a sample) and abnormal trace element bioaccumulation (based on hypothesized background and toxicity levels in the relevant literature) [36,45,46,47,48,49,50]. Slides were reviewed by a board-certified veterinary pathologist for histopathologic changes that included, but were not limited to, the presence of pathogens, neoplasia, inflammation, and cellular alterations (e.g., hyperplasia, degeneration, and necrosis). Histopathology reports were reviewed for anomalies that may be associated with MC exposure or trace element toxicosis, including hepatocellular or renal necrosis, fibrosis, hemorrhage, inflammation, or lipidosis/vacuolation, hepatic extramedullary hematopoiesis (EMH), primary liver cancer, renal tubular degeneration or congestion, and/or glomerulosclerosis [24,30,50,51,52,53,54,55,56,57,58,59,60]. 

### 2.6. Statistical Evaluation 

Arithmetic means and standard deviations were calculated for each trace element in both tissue types using Microsoft® Excel® for Microsoft 365 MSO (Version 2409 Build 16.0.18025.20030) 64-bit. A Mantel test was performed using the “vegan” package (R Core Team, 2024) to determine if liver and kidney trace element values were correlated. Single-factor Permutational Multivariate Analysis of Variances (PERMANOVAs) were performed using the “vegan” package (R Core Team, 2024) to evaluate if element concentrations in the liver and kidney were dependent on sex, season, age class, or year [61]. As previous studies in other free-ranging North American river otter populations have demonstrated sex-related differences in liver Cu, Mo, and Hg levels [46,47], these specific trace elements were analyzed by one-way Analysis of Variance (ANOVA) to determine if similar patterns were present. *p*-values equal to or less than 0.05 were considered statistically significant. The trace element concentrations were transformed if needed (log or square root + 1) to meet test assumptions for normality and homogeneity of variance [62]. Element concentrations were also normalized for the PERMANOVA and Mantel tests as the data were measured on different scales [61]. Prior to the one-way ANOVAs, pre-hoc tests were performed to test for assumptions, including a Shapiro–Wilks test for normality and a Bartlett’s test for homogeneity of variance [62].

## 3. Results

### 3.1. Study Animals

**Figure 1:**
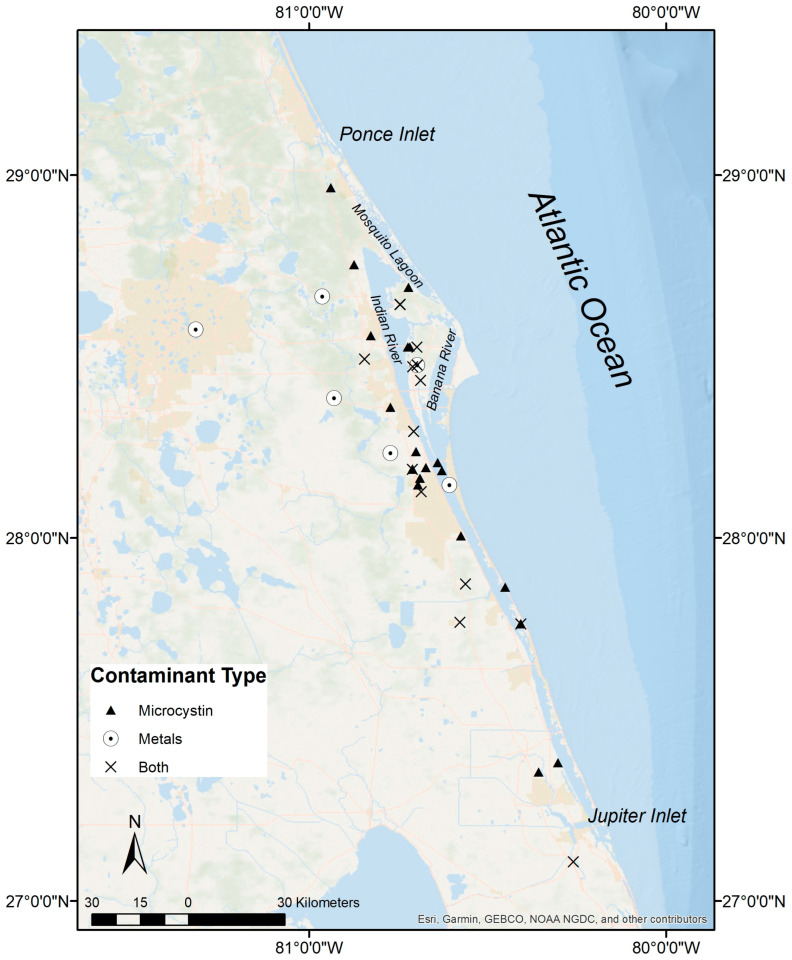
Carcass recovery locations (2016–2022) of Indian River Lagoon (IRL) North American river otters (*Lontra canadensis*) screened for contaminant exposure.

A total of forty-three river otters were evaluated in this study. Age class was determined for 98% (*n* = 42) of individuals and consisted of 93% adults (*n* = 39) and 7% juveniles (*n* = 3) (Table S1). Total body length was measured for 98% (*n* = 42) of individuals and ranged from 74 to 125 cm (Table S1). Forty-two percent of river otters were female (*n* = 18) and 58% were male (*n* = 25) (Table S1). Carcass recovery locations ranged from Edgewater, Volusia County (28.965262 N, −80.938802 W) in the north to Palm City, Martin County (27.108923 N, −80.25897 W) in the south (Table S1; Figure 1). Most river otters (84%, *n* = 36) were observed in the northern IRL watershed. Vehicle trauma was the cause of mortality in 95% (*n* = 41) of cases (Table S1).

### 3.2. Microcystin Exposure

Most river otters (86%, *n* = 37) were evaluated for MC exposure using liver samples collected between May 2016 and May 2022. Microcystin was not detected in any of the river otter liver samples analyzed by enzyme immunoassay (ELISA) (*n* = 37) or by MMPB (*n* = 4) (Table S2). The majority of river otters (81%, *n* = 30) screened for MC exposure were observed in the northern IRL watershed.

### 3.3. Trace Element Exposure

Approximately half of river otters (52%, *n* = 22) were evaluated for trace element bioaccumulation using paired liver and kidney samples collected between May 2016 and August 2018 (Tables S2–S4, Table 1 and Table 2). Tl was the only element not detected in the liver or kidney of any individual and was, therefore, not included in statistical analysis. As expected, six of the essential elements (Cu, Fe, Mn, Mo, Se, and Zn) were detected in all individuals. The limit of quantification (LOQ) was not available from MSU for these elements. Most river otter samples were below the LOQ in the liver and kidney for As, as well as in the kidney only for Co. Average concentrations were higher in the liver compared to the kidney for most trace elements tested (Co, Cu, Fe, Hg, Mn, Mo, Pb, and Zn), while average values were higher in kidney for As, Cd, and Se. The dry matter fraction was above the reference interval (0.19–0.24 = kidney; 0.26–0.34 = liver) for 95% (*n* = 21) of kidney samples and 4% (*n* = 1) of liver samples (Tables S3 and S4). No sample measured above the reference ranges provided by MSU (As ≤ 9.00 μg/g dw; Cd ≤ 600.00 μg/g dw; Hg ≤ 30.00 μg/g dw; and Pb ≤ 3.00 μg/g dw) or the estimated toxic threshold based on similar studies [36,45,46,47,48,49,50].

The results of the Mantel test demonstrated that liver and kidney trace element values were not correlated (Mantel statistic r = -0.069, *p* = 0.679). Of the river otters tested for trace element bioaccumulation, 41% (*n* = 9) were female and 59% (*n* = 13) were male. The results of single-factor PERMANOVA tests demonstrated that sex did not explain the variance between trace element levels in the liver (Table 3) but did explain approximately 10% of the variance in the kidney (Table 4). In contrast to similar studies, there were no statistically significant differences based on sex between liver Cu, Hg, or Mo values as determined by one-way ANOVA tests (Table 5, Table 6 and Table 7). Notably, the highest individual levels were detected in females for all elements except liver Cu, Fe, Mn, Mo, and Pb, and kidney As and Fe.

The season and year carcass recovery occurred were available for 100% (*n* = 22) of river otters tested for trace element bioaccumulation (Table S1). Most of the river otter carcasses were recovered in the winter (32%, *n* = 7), followed by spring (27%, *n* = 6), fall (23%, *n* = 5), and summer (18%, *n* = 4). The year of carcass recovery included 23% (*n* = 5) in 2016, 63.5% (*n* = 14) in 2017, and 13.5% (*n* = 3) in 2018. Body length for these individuals ranged from 79 to 125 cm and consisted of 96% (*n* = 21) adults and 4% (*n* = 1) juveniles. Statistical analysis of the impact of season, year, or age class could not be performed due to unequal sample sizes for predictor variable groups.

One river otter was undergoing rehabilitation at a wildlife hospital for over four months prior to dying from natural causes. Due to the limited sample size, statistical comparisons could not be made between this individual and those that were not under human-managed care. However, this individual demonstrated the highest liver and kidney Zn, lowest kidney Se, and second lowest liver and kidney Hg levels, in this study.

### 3.4. Health Analysis

Liver and kidney histopathology were evaluated for 65% (*n* = 28) of river otters (Table S2). Of those, 79% (*n* = 22) were screened for MC exposure and 71% (*n* = 20) for trace element bioaccumulation. 

Eighteen percent (*n* = 5) of river otters had liver anomalies potentially consistent with MC exposure or abnormal trace element bioaccumulation, including non-specific inflammation, necrosis, and lipidosis (Table S2 and Table 8). Inflammation ranged from mild (25%, *n* = 1) to moderate (75%, *n* = 3) and focal (25%, *n* = 1) to multifocal (75%, *n* = 3) and was characterized as lymphoplasmacytic (*n* = 2), histiocytic (*n* = 1), granulomatous (*n* = 1), necrotizing (*n* = 1), and/or mixed inflammatory (*n* = 1) (Tables S2 and S5; Figures S1–S4). The lipidosis was multifocal and suspect (Tables S2 and S5; Figure S5). Other liver findings were traumatic fracture (*n* = 5) and nodular hyperplasia (*n* = 2) (Table S2; Figure S5). The hyperplasia ranged from focal (50%, *n* = 1) to multifocal (50%, *n* = 1) (Table S5). Liver dysfunction was suspected in two river otters with hepatic inflammation and/or necrosis but did not appear to be the cause of substantial morbidity or mortality (Table S2). Since MC and abnormal trace element bioaccumulation were not detected in this study, these liver lesions were not linked to contaminant exposure. 

Kidney lesions were observed in 7% (*n* = 2) of river otters (Table S2 and Table 9). Kidney lesions were only observed in adult males (Tables S1 and S2). One river otter demonstrated a renal lesion (i.e., a mild, multifocal non-specific lymphoplasmacytic inflammation) that could be caused by contaminant exposure (Tables S2 and S5; Figure S6). Medullary dilation and mineralization (mild, focal) were observed in another individual (Tables S2 and S5; Figure S7). No river otter in this study had apparent renal dysfunction. Since MC and abnormal trace element bioaccumulation were not detected in this study, kidney lesions were not linked to contaminant exposure in IRL river otters. 

Statistical analysis of the impact of sex, season, year, or age class on the presence of liver or kidney lesions could not be performed due to unequal sample sizes for predictor variable groups.

## 4. Discussion

### 4.1. Microcystin Exposure

The present study represents the first systematic survey for a HAB toxin in IRL river otters. Microcystin exposure and related toxicity were not detected in the IRL river otter population. However, MC has been previously detected in 5–7.5% of rehabilitating green sea turtles and 20% of free-ranging bull sharks from the IRL [3,8]. River otters in the IRL may avoid MC exposure due to their prey selection (low or no MC) or geographic distribution, or they be exposed to sub-detectable levels. The diet and movement patterns of river otters specific to the IRL have not been described, but finfish and invertebrates are typically the most important prey items for these opportunistic foragers [63,64]. Vectors of MC to IRL megafauna are also unknown, though striped mullet (*Mugil cephalus*) was hypothesized [3]. Habitat usage and movement patterns of IRL river otters and their prey require investigation. Seasonal changes in temperature and rainfall are known to impact other river otter populations [65]. Most river otters in this study were found in the northern IRL watershed, whereas MC is more commonly detected in the southern IRL [23,25,26,27,28]. Thus, the subpopulations most at risk for MC exposure may not be adequately represented herein. Although MC exposure or toxicity has not been reported in river otters to date, southern sea otters in central California have succumbed to acute MC toxicosis after contaminated bivalve ingestion [30]. This suggests that IRL river otters are also susceptible. 

Although MC-induced acute liver toxicosis was not detected in IRL river otters in this study, long-term, low-level exposure may have contributed to the liver and kidney lesions observed as the toxin may have no longer been present in the body or been so at sub-detectable levels. Microcystin exposure doses and subsequent tissue levels and lesions that correspond to acute and chronic disease in aquatic mammals are unknown, but total hepatic levels as high as 14.3 ± 5.6 ng/g were not associated with overt toxicosis in estuarine bottlenose dolphins in northeast Florida [66]. Similarly, liver concentrations as high as 82.5 ng/g and 100.2 ng/g were not associated with obvious toxicosis in IRL bull sharks or green sea turtles, respectively [3,8]. Future studies evaluating MC loads in river otters inhabiting highly contaminated regions (i.e., Lake Okeechobee) may help elucidate background exposure versus toxic thresholds. 

### 4.2. Trace Element Exposure

The detection of six essential elements (Cu, Fe, Mn, Mo, Se, and Zn) in every individual in this study was not surprising as they are all required for healthy bodily function [67]. The elements that tested below the LOQ for one or more samples submitted mimicked findings in another river otter population in the southeast U.S. [46]. The average trace element concentrations detected in the liver and kidney of IRL river otters generally followed similar trends as IRL bottlenose dolphins and manatees, as well as other river otter populations in the continental U.S. [16,19,36,46,48,67]. Hg, Se, and Zn levels in IRL bottlenose dolphin liver samples were substantially higher than those observed in IRL river otters, while Cu levels were higher in river otters [17,19]. The same trend for Hg, Se, and Zn was observed between estuarine bottlenose dolphins and river otters in Charleston, SC, suggesting there may be species-specific dietary or biological factors influencing bioaccumulation of these trace elements, such as differences in foraging behaviors, prey preference, or trace element absorption, metabolism, storage, or elimination [17,68]. For example, many of the primary prey of IRL bottlenose dolphins, such as spotted sea trout (*Cynoscion nebulosus*), silver perch (*Bairdiella chrysoura*), Atlantic croaker (*Micropogonias undulates*), and striped mullet, are known to bioaccumulate Hg [20]. As opportunists, IRL river otters may consume prey with little to no Hg, or their movement patterns may not spatiotemporally overlap with highly contaminated regions. Unlike bottlenose dolphins, river otters typically consume some terrestrial prey, which may contain lower Hg levels compared to aquatic organisms [36]. Most river otters were found in the northern half of the IRL, whereas most of the nutrient run-off may be concentrated in the southern half [69]. River otters also have a rapid gastrointestinal (GI) transit time that may prevent the complete absorption of some trace elements into the systemic circulation [70]. Hg, Se, and Zn levels in IRL river otters likely represent background exposure as they were below the toxic threshold, not associated with histopathological anomalies, and, in the case of Hg and Se, comparable to values observed in other southeast U.S. river otter populations [46,71]. The average liver Hg level in IRL river otters was lower than the proposed background level (4 μg/g dw) for the species [45,49]. However, since high-level Hg bioaccumulation and associated liver anomalies have been observed in IRL bottlenose dolphins at levels known to be toxic to river otters and other mammals, and Hg has been suspected of impacting the stability of other river otter populations worldwide [17,18,19,21,45,50], continued Hg monitoring is recommended in the IRL population whenever feasible. 

River otters in this study demonstrated higher average Cu concentrations than IRL bottlenose dolphins and other river otter populations in the continental U.S. [19,36,45,46,48,67,72], suggesting both geographic and species-specific influences. Cu is an essential element that typically declines with age [31]. Proximity to Cu-containing terrestrial run-off, fertilizers, pesticides, algicides, and anti-fouling paints used to protect ship hulls in the lagoon may have contributed to higher exposure in IRL river otters [32,73]. Notably, IRL finfish and invertebrates also demonstrate high Cu levels from pollutant stress and may have vectored the element up the food web [32,74]. Cu exposure could have been further magnified due to the river otter’s high daily dietary intake [70]. High Cu levels may also be due to differential Cu absorption, metabolism, storage, and elimination based on population or species [75]. Higher Cu levels were also observed in manatees in the IRL and other urban areas, as well as those in captivity presumptively due to romaine lettuce intake [16]. A Cu kidney value (52 μg/g dw) in one IRL river otter was close to the toxic level for sensitive species [76,77]. However, there was no evidence of toxic pathology, including deposition of pigment within hepatocytes or renal tubular epithelial cells. Cu poisoning was suspected in a dugong (*Dugong dugon*) in eastern Australia from exposure to an algicide [78], but has never been reported in a river otter or other marine mammal. Normal ranges of Cu in the liver and kidney of river otters, as well as the toxic dose and lesions, are unknown. River otters may be able to tolerate higher Cu levels than highly sensitive species as they have a reduced sensitivity to some other environmental contaminants [36]. Eurasian otters (*Lutra lutra*) have demonstrated Cu levels similar to this study with no overt anomalies [45,72]. Though obvious toxicity (e.g., cirrhosis, GI bleeding) was not apparent in this study [31], IRL river otters may experience subtle health impacts from Cu bioaccumulation, particularly concurrent with other natural or anthropogenic stressors [45]. 

In addition to Cu, other differences in trace element bioaccumulation were observed between the IRL and other river otter populations. Compared to other river otters in the continental U.S., those in the IRL also had higher Fe and Zn levels [46,48]. This may be due to differential Fe and Zn bioaccumulation in prey from natural or anthropogenic inputs or immune perturbations causing sequestration of the elements in IRL river otters [31,61]. The difference in Fe levels may also represent an artifact from postmortem accumulation of red blood cells (RBCs) in the liver and kidneys. Little is known about Fe accumulation in IRL prey but irrigation water and soil run-off could account for the increased exposure to IRL river otters [79]. Bottlenose dolphins in the IRL also demonstrated higher Fe levels than those from South Carolina [19]. Notably, IRL finfish have previously been observed to have high levels of Zn, likely from pollutant stress [74]. However, Fe and Zn levels were still well below toxic thresholds and are not of pressing concern to the health of IRL river otters. 

Differential element bioaccumulation between the liver and kidney of IRL river otters followed similar trends as previously reported in other North American populations and species [46,49,80]. Consistent with this study, average Co, Cu, Fe, Hg, Mn, Mo, Pb, and Zn were all higher in the liver compared to the kidney in North Carolina river otters [46]. However, there were differences in relative organ concentrations of As, Cd, and Se between the IRL and North Carolina populations. This may be due to differences in age, body size, or underlying health of the study populations. However, consistent with this study, Cd is typically higher in the kidney, while contrary to this study, Se is usually higher in the liver of mammals [49,81]. Also consistent with this study, higher average liver Hg values are typically observed in other marine mammal species [61]. 

There were no statistically significant patterns observed based on sex, season, year, or age class in this study except that sex had a small influence on trace element levels in the kidney. Few sex-related differences in trace element levels have been reported in other river otter populations. Female river otters in North Carolina demonstrated higher liver Cu and lower Mo levels than males, while Hg levels were higher in female river otters in eastern Canada [46,47]. No such patterns were observed in our study, though the highest liver values of Cu and Mo were both in males and the highest Hg value was in a female. Sex-related differences in trace element levels were not detected in manatees [16], but were observed in bottlenose dolphins [17]. The impact of season, year, and age class could not be evaluated due to small and unequal sample sizes. However, population-specific age-related differences in trace element bioaccumulation have been observed in other river otter populations [36,46], as well as in manatees and bottlenose dolphins [16,17,19]. While bioaccumulation increased with age for certain trace elements in North Carolina river otters (i.e., Cd, Fe, Hg, Mo, and Se) and bottlenose dolphins (i.e., Hg, Pb, and Se), age-related decreases were also observed in manatees (i.e., Cd, Cu, Pb, and Tl) and bottlenose dolphins (i.e., Mn) [16,46]. Age and sex-related differences in tissue trace element bioaccumulation may result from differential prey selection or element absorption, metabolism, storage, or elimination [17]. Element offloading in pregnant or lactating females may also contribute to sex-based differences [17]. Although not detected in our study, free-ranging pinnipeds may exhibit seasonal variations in trace element levels [82]. A larger sample size may help elucidate sex, seasonal, and age-related differences in trace element bioaccumulation in IRL river otters.

No other studies have evaluated differential trace element levels in rehabilitating versus free-ranging river otters. Higher Zn and lower Hg and Se levels were noted in this study, but the low sample size precluded statistical analysis. Florida manatees undergoing rehabilitation had lower whole blood Se and Zn levels than free-ranging populations [16]. Such differences may be related to nutritional supplementation or diet during rehabilitation, as well as underlying health status. For example, the lower Se and higher Zn levels in the rehabilitating river otter may have resulted from the immobilization or sequestration of these essential elements to aid in inflammation, nutrition, and healing [16]. Conversely, most of the other river otters in this study appeared relatively healthy as the major cause of mortality was vehicle trauma. 

Of note, kidney element concentrations may have been impacted as the dry matter fractions were above the reference interval either due to patient dehydration or fatty infiltration. Nevertheless, the trace element contents of IRL river otters were not near presumed toxic thresholds for the species and were generally similar to those reported in other river otter populations [36,45,46,47,48,49,50,67]. Thus, the trace element concentrations reported herein likely represent background levels. There was no evidence of trace element toxicity in IRL river otters as liver and kidney lesions were not linked to high-level exposure.

### 4.3. Health Analysis

Microcystin exposure or trace element bioaccumulation did not cause morbidity or mortality in IRL river otters. Other differentials (e.g., current or previous protozoal, viral, or bacterial infection, metabolic disease, and other anthropogenic contaminants) should be considered for the liver and kidney lesions observed in this study [37]. For example, exposure to pollutant petroleum products can cause liver inflammation in free-ranging river otters [37]. Free-ranging river otters are also at risk for developing uric acid nephrolithiasis [83], which may have caused the kidney mineralization and dilation observed. Primary liver or kidney disease did not contribute to mortality or substantially impact the fitness or survival of IRL river otters. However, the presence of liver or kidney lesions may increase the vulnerability of these animals to contaminant exposure in the future [84].

## 5. Conclusions

Microcystin was not detected in river otters in the IRL estuary system. Trace elements were present at presumed sub-toxic levels and likely represent background exposure. While Cu was detected at concentrations that may cause toxicity in sensitive species, it did not induce overt disease in IRL river otters, suggesting this species may have a higher tolerance. Though the IRL river otter population experiences liver and kidney insult, lesions were not linked with MC exposure or atypical trace element bioaccumulation. Other causes (e.g., infectious agents, metabolic disease) should be considered for the liver and kidney lesions observed in this study. Green sea turtles and/or bull sharks may be more appropriate sentinels of MC exposure in the IRL due to their foraging, habitat use, or other life history characteristics. While river otters may be valuable sentinels of Cu exposure in the IRL, bottlenose dolphins may provide better insight into the severity of Hg, Se, and Zn biomagnification in the system. Continued contaminant screening is recommended whenever feasible in IRL river otters to better establish background versus toxic exposure levels, while alerting the public to emerging ecosystem-wide threats. Future research is also recommended characterizing the movement patterns and prey preferences specific to river otters in the IRL to determine sources of contaminant exposure in this sentinel species. 

## Figures and Tables

**Table 1:** The concentration of trace elements measured by inductively coupled plasma mass spectrometry (ICP-MS) in the liver of deceased North American river otters (*n* = 22) inhabiting the IRL watershed (2016–2018). Values are reported in μg/g dry weight (dw). Limit of quantification = LOQ; NR = not reported by the laboratory; SD = standard deviation; ND = not detected.

Element	LOQ	Otters above LOQ	Detection Range	Mean ± SD
As	<0.08	14% (*n* = 3)	ND–0.31	0.03 ± 0.09
Cd	<0.08	73% (*n* = 16)	ND–0.68	0.18 ± 0.18
Co	<0.04	59% (*n* = 13)	ND–0.09	0.03 ± 0.03
Cu	NR	100% (*n* = 22)	15.9–149.6	42.3 ± 28.3
Fe	NR	100% (*n* = 22)	136–1565	591 ± 386
Hg	<0.41	96% (*n* = 21)	ND–8.38	2.2 ± 1.72
Mn	NR	100% (*n* = 22)	5.3–21.3	10.8 ± 3.5
Mo	NR	100% (*n* = 22)	1.68–4.15	2.74 ± 0.6
Pb	<0.08	59% (*n* = 13)	ND–0.54	0.13 ± 0.15
Se	NR	100% (*n* = 22)	1.51–3.21	2.35 ± 0.4
Tl	<0.08	0%	ND	ND
Zn	NR	100% (*n* = 22)	69.4–171	92.2 ± 20.6

**Table 2:** The concentration of trace elements measured by ICP-MS in the kidney of deceased North American river otters (*n* = 22) inhabiting the IRL watershed (2016–2018). Values are reported in μg/g dw. Limit of quantification = LOQ; NR = not reported by the laboratory; SD = standard deviation; ND = not detected.

Element	LOQ	Otters above LOQ	Detection Range	Mean ± SD
As	<0.12	14% (*n* = 3)	ND–0.52	0.05 ± 0.14
Cd	<0.12	91% (*n* = 20)	ND–3.51	0.82 ± 0.9
Co	<0.06	14% (*n* = 3)	ND–0.06	0.006 ± 0.017
Cu	NR	100% (*n* = 22)	6.3–52	14.4 ± 8.9
Fe	NR	100% (*n* = 22)	171–754	344 ± 154
Hg	<0.61	96% (*n* = 21)	ND–6.57	2.08 ± 1.55
Mn	NR	100% (*n* = 22)	0.84–9.8	2.8 ± 1.7
Mo	NR	100% (*n* = 22)	0.3–2.2	0.79 ± 0.35
Pb	<0.12	59% (*n* = 10)	ND–0.4	0.08 ± 0.12
Se	NR	100% (*n* = 22)	2.86–5	4.1 ± 0.69
Tl	<0.12	0%	ND	ND
Zn	NR	100% (*n* = 22)	35.5–105	72.1 ± 13.2

**Table 3:** A single-factor PERMANOVA evaluating the impact of sex on trace element concentrations in the liver of IRL river otters (total *n* = 22; females, *n* = 9; males, *n* = 13). Data analyzed after square root + 1 transformation and normalization of the trace element values.

	Df	Sum of Squares	R^2^	F	*p*-Value
**Sex**	1	16.324	0.071	1.521	0.125
**Residual**	20	214.676	0.929		
**Total**	21	231	1		

**Table 4:** A single-factor PERMANOVA evaluating the impact of sex on trace element concentrations in the kidney of IRL river otters (total *n* = 22; females, *n* = 9; males, *n* = 13). Data analyzed after square root + 1 transformation and normalization of the trace element values.

	Df	Sum of Squares	R^2^	F	*p*-Value
**Sex**	1	22.238	0.096	2.131	0.027
**Residual**	20	208.762	0.904		
**Total**	21	231	1		

**Table 5:** A 1-way ANOVA evaluating the impact of sex on liver Cu values of IRL river otters (total *n* = 22; females, *n* = 9; males, *n* = 13). Data analyzed with a log transformation of the Cu values.

	Df	Sum of Squares	Mean Square	F	*p*-Value
**Sex**	1	0.162	0.163	0.578	0.456
**Residual**	20	5.625	0.281		
**Total**	21	5.237	0.444		

**Table 6:** A 1-way ANOVA evaluating the impact of sex on liver Hg values of IRL river otters (total *n* = 22; females, *n* = 9; males, *n* = 13). Data analyzed after square root + 1 transformation of the Hg values.

	Df	Sum of Squares	Mean Square	F	*p*-Value
**Sex**	1	0.491	0.490	2.926	0.103
**Residual**	20	3.353	0.168		
**Total**	21	3.844	0.658		

**Table 7:** A 1-way ANOVA evaluating the impact of sex on liver Mo values of IRL river otters (total *n* = 22; females, *n* = 9; males, *n* = 13). Transformation of the Mo values was not necessary to meet test assumptions.

	Df	Sum of Squares	Mean Square	F	*p*-Value
**Sex**	1	0.455	0.455	1.304	0.267
**Residual**	20	6.980	0.349		
**Total**	21	7.435	0.804		

**Table 8:** Types and incidence of histologic liver lesions observed in deceased IRL river otters between 2016 and 2022. Lesions potentially consistent with MC or trace element toxicity are in italics.

Liver Lesion	Incidence in IRL River Otters
*Inflammation*	14% (*n* = 4)
*Necrosis*	7% (*n* = 2)
*Lipidosis*	4% (*n* = 1)
Traumatic fracture	18% (*n* = 5)
Nodular hyperplasia	7% (*n* = 2)

**Table 9:** Types and incidence of histologic renal lesions observed in deceased IRL river otters between 2016 and 2022. Lesions potentially consistent with MC or trace element toxicity are in italics.

Renal Lesion	Incidence in IRL River Otters
*Inflammation*	4% (*n* = 1)
Medullary Dilation	4% (*n* = 1)
Mineralization	4% (*n* = 1)

## Data Availability

The original contributions presented in the study are included in the article/Supplementary Material, further inquiries can be directed to the corresponding author/s.

## Supplementary Materials

The following supporting information can be downloaded at: https://www.mdpi.com/article/10.3390/toxics12090684/s1, Figure S1: Moderate, multifocal hepatitis (lymphoplasmacytic and histiocytic) and hepatocellular necrosis/loss (arrow) in an IRL river otter (MKS-1704-Lc); H & E, 300 dpi, 40×, scale bar = 20 µm. Image credit/Histologic interpretation: Dr. David Rotstein; Figure S2: Mild-moderate, multifocal, active hepatitis (necrotizing and mixed inflammatory) in an IRL river otter (MKS-1706-Lc); H & E, 300 dpi, 20×. Image credit/Histologic interpretation: Dr. David Rotstein; Figure S3: Focal portal hepatitis (lymphoplasmacytic) in an IRL river otter (MKS-1724-Lc); H & E, 300 dpi, 20×. Image credit/Histologic interpretation: Dr. David Rotstein; Figure S4: Mild-moderate multifocal hepatitis (granulomatous) in an IRL river otter (MKS-2206-Lc); H & E, 300 dpi, 20×. Image credit/Histologic interpretation: Dr. David Rotstein; Figure S5: Multifocal hepatic lipidosis (suspect) and focal nodular hyperplasia in an IRL river otter (MKS-1720-Lc); H & E, 300 dpi, 40×. Image credit/Histologic interpretation: Dr. David Rotstein; Figure S6: Mild, multifocal interstitial nephritis (lymphoplasmacytic) in an IRL river otter (MKS-2117-Lc); H & E, 300 dpi, 20×. Image credit/Histologic interpretation: Dr. David Rotstein; Figure S7: Mild, focal medullary dilation and mineralization in the kidney of an IRL river otter (MKS-1724-Lc); H & E, 300 dpi, 10×. Image credit/Histologic interpretation: Dr. David Rotstein. Table S1: Raw data for Indian River Lagoon (IRL) North American river otters (Lontra canadensis) (*n* = 43) evaluated for microcystin (MC) and/or trace element bioaccumulation (2016–2022), including carcass recovery location, date, and season, and demographic information. N/A = data not available; Table S2: Raw data for IRL river otters (*n* = 43) evaluated for MC and/or trace element bioaccumulation (2016–2022), including MC liver values, liver and kidney histopathology results, and if trace element analysis was performed. See Tables S3 and S4 for trace element levels in the liver and kidney. N/A = data or samples not available, MDL = minimum detection limit, MMPB = 2-methyl-3-methoxy-4-phenylbutyric acid technique, NSF = no significant histopathological findings; Table S3: Trace element levels (μg/g dry weight [dw]) in the liver of IRL river otters (*n* = 22) measured by inductively coupled plasma mass spectrometry (ICP-MS) (2016–2018); Table S4: Trace element levels (μg/g dw) in the kidney of IRL river otters (*n* = 22) measured by ICP-MS (2016–2018); Table S5: The severity of microscopic inflammation in IRL river otters evaluated for MC and/or trace element bioaccumulation (2016–2022) with non-traumatic liver and/or kidney histopathologic anomalies (*n* = 7). NSF = no significant histopathological findings, + = mild inflammation, ++ = moderate inflammation, +++ = severe inflammation.
