# Supplementary material for: Contaminant Exposure and Liver and Kidney Lesions in North American River Otters in the Indian River Lagoon, Florida
Source: Toxics. 2024 Sep 21;12(9):684. doi: 10.3390/toxics12090684 (PMC11435442; doi:10.3390/toxics12090684)
Supplement: Supplementary file 1 [file toxics-12-00684-s001.zip › toxics-3137146-supplementary.pdf]

Table S1. Raw data for Indian River Lagoon (IRL) North American river otters (*Lontra canadensis*) (*n* = 43) evaluated for microcystin (MC) and/or trace element bioaccumulation (2016-2022), including carcass recovery location, date, and season, and demographic information. N/A = data not available

| FIT ID | Sender ID | Latitude | Longitude | Season | Stranding date | Decomp code | Body length (cm) | Age class | Sex | Vehicle trauma |
|---|---|---|---|---|---|---|---|---|---|---|
| IRLC015 | MKS-1603-Lc | 28.476742 | -80.696208 | Spring | 5/18/2016 | 2 | 125 | Adult | Male | Yes |
| IRLC044 | MKS-1604-Lc | 28.476742 | -80.696208 | Spring | 5/18/2016 | 2 | 114 | Adult | Male | Yes |
| N/A | MKS-1605-Lc | 28.476742 | -80.696208 | Spring | 5/18/2016 | 2 | 107 | Adult | Male | Yes |
| IRLC008 | MKS-1607-Lc | 28.293678 | -80.706703 | Summer | 6/28/2016 | 3 | 98 | Adult | Female | Yes |
| IRLC037 | MKS-1610-Lc | 28.643555 | -80.74518 | Winter | 12/2/2016 | 2 | 117 | Adult | Male | Yes |
| IRLC042 | MKS-1702-Lc | 27.763303 | -80.406216 | Winter | 1/8/2017 | 2 | 102 | Adult | Male | Yes |
| IRLC020 | MKS-1704-Lc | 28.643554 | -80.744171 | Winter | 1/25/2017 | 2 | 100 | Adult | Female | Yes |
| IRLC041 | MKS-1706-Lc | 28.293773 | -80.706593 | Winter | 2/23/2017 | 2 | 107 | Adult | Female | Yes |
| N/A | MKS-1708-Lc | 28.385824 | -80.929686 | Spring | 3/16/2017 | 2 | 98 | Adult | Female | Yes |
| N/A | MKS-1711-Lc | 28.146672 | -80.606046 | Spring | 4/27/2017 | 2 | 79 | Juvenile | Male | No |
| IRLC032 | MKS-1712-Lc | 27.873533 | -80.56164 | Spring | 5/16/2017 | 2 | 108 | Adult | Female | Yes |
| N/A | MKS-1716-Lc | 28.234205 | -80.771321 | Summer | 8/14/2017 | 2 | N/A | Adult | Female | No |
| IRLC039 | MKS-1718-Lc | 28.127741 | -80.685275 | Fall | 10/6/2017 | 2 | 109 | Adult | Male | Yes |
| IRLC023 | MKS-1719-Lc | 28.472406 | -80.709362 | Fall | 11/2/2017 | 2 | 117 | Adult | Male | Yes |
| N/A | MKS-1720-Lc | 28.574642 | -81.317104 | Fall | 11/5/2017 | 2 | 107 | Adult | Male | Yes |
| IRLC017 | MKS-1722-Lc | 27.108923 | -80.25897 | Fall | 11/15/2017 | 2 | 97 | Adult | Female | Yes |
| IRLC006 | MKS-1724-Lc | 28.526378 | -80.697617 | Fall | 11/20/2017 | 3 | 120 | Adult | Male | Yes |

| | | | | | | | | | | |
|---|---|---|---|---|---|---|---|---|---|---|
| IRLC028 | MKS-1725-Lc | 28.189322 | -80.709863 | Winter | 12/4/2017 | 3 | 100 | Adult | Female | Yes |
| IRLC009 | MKS-1727-Lc | 28.43433 | -80.686924 | Winter | 12/21/2017 | 3 | 120 | Adult | Male | Yes |
| N/A | MKS-1802-Lc | 28.665945 | -80.962697 | Winter | 1/26/2018 | 3 | 106 | Adult | Male | Yes |
| IRLC045 | MKS-1811-Lc | 28.752704 | -80.873216 | Summer | 6/11/2018 | 3 | 107 | Adult | Female | Yes |
| IRLC030 | MKS-1813-Lc | 27.767619 | -80.577059 | Summer | 7/1/2018 | 2 | 101 | Adult | Female | Yes |
| IRLC034 | MKS-1815-Lc | 28.4937 | -80.8439 | Summer | 8/26/2018 | 3 | 119 | Adult | Male | Yes |
| IRLC011 | MKS-1817-Lc | 27.3555 | -80.3567 | Fall | 11/30/2018 | 3 | 100 | Adult | Female | Yes |
| IRLC013 | MKS-1818-Lc | 27.863998 | -80.450392 | Winter | 12/6/2018 | 2 | 113 | Adult | Male | Yes |
| IRLC025 | MKS-1819-Lc | 28.20746 | -80.639614 | Winter | 12/13/2018 | 3 | 115 | Adult | Male | Yes |
| IRLC010 | MKS-1901-Lc | 28.5264 | -80.7233 | Winter | 1/7/2019 | 3 | 116 | Adult | Male | Yes |
| IRLC029 | MKS-1902-Lc | 28.5265 | -80.7176 | Winter | 1/7/2019 | 2 | 110 | Adult | Female | Yes |
| IRLC040 | MKS-1910-Lc | 27.763335 | -80.407074 | Winter | 1/26/2019 | 3 | 107 | Adult | Male | Yes |
| IRLC046 | MKS-1921-Lc | 28.00548 | -80.574521 | Spring | 5/22/2019 | 3 | 106 | Adult | Male | Yes |
| IRLC033 | MKS-2007-Lc | 28.190183 | -80.709863 | Spring | 3/20/2020 | 2 | 120 | Adult | Female | Yes |
| IRLC026 | MKS-2009-Lc | 28.165022 | -80.688957 | Spring | 4/9/2020 | 2 | 109 | Adult | Female | Yes |
| IRLC035 | MKS-2011-Lc | 28.557684 | -80.826792 | Fall | 11/3/2020 | 3 | 108 | Adult | Female | Yes |
| IRLC007 | MKS-2012-Lc | 28.18552 | -80.628015 | Fall | 11/30/2020 | 4 | 109 | Adult | Male | Yes |
| IRLC016 | MKS-2015-Lc | 28.14615 | -80.6944 | Winter | 12/17/2020 | 3 | 113 | Adult | Male | Yes |
| IRLC049 | MKS-2102-Lc | 27.7635 | -80.40606 | Winter | 1/12/2021 | 2 | 114 | Adult | Male | Yes |

| | | | | | | | | | | |
|---|---|---|---|---|---|---|---|---|---|---|
| IRLC047 | MKS-2110-Lc | 27.3813258 | -80.30271 | Winter | 12/8/2021 | 3 | 80 | Juvenile | Female | Yes |
| IRLC019 | MKS-2116-Lc | 28.690596 | -80.721474 | Winter | 12/2/2021 | 3 | 108 | Adult | Male | Yes |
| IRLC048 | MKS-2117-Lc | 28.19369 | -80.672174 | Winter | 12/4/2021 | 3 | 104 | Adult | Male | Yes |
| IRLC021 | MKS-2201-Lc | 28.189561 | -80.709844 | Winter | 1/13/2022 | 3 | 107 | Adult | Male | Yes |
| IRLC043 | MKS-2206-Lc | 28.360093 | -80.771569 | Spring | 3/15/2022 | 3 | 106 | Adult | Male | Yes |
| IRLC051 | MKS-2212-Lc | 28.965262 | -80.938802 | Spring | 3/29/2022 | 3 | 74 | Juvenile | Female | Yes |
| IRLC038 | MKS-2215-Lc | 28.23773 | -80.7001 | Spring | 5/25/2022 | 2 | 97 | Adult | Female | Yes |

Table S2. Raw data for IRL river otters ($n$ = 43) evaluated for MC and/or trace element bioaccumulation (2016-2022), including MC liver values, liver and kidney histopathology results, and if trace element analysis was performed. See Tables S3 and S4 for trace element levels in the liver and kidney. N/A = data or samples not available, MDL = minimum detection limit, MMPB = 2-methyl-3-methoxy-4-phenylbutyric acid technique, NSF = no significant histopathological findings

| **Sender ID** | **MC ELISA results (ng/g)** | **MC MMPB results (ng/g)** | **Trace elements tested** | **Liver histopathology** | **Kidney histopathology** |
|---|---|---|---|---|---|
| MKS-1603-Lc | < MDL | N/A | Yes | NSF | NSF |
| MKS-1604-Lc | < MDL | N/A | Yes | NSF | NSF |
| MKS-1605-Lc | N/A | N/A | Yes | NSF | NSF |
| MKS-1607-Lc | < MDL | N/A | Yes | NSF | NSF |
| MKS-1610-Lc | < MDL | N/A | Yes | NSF | NSF |
| MKS-1702-Lc | < MDL | N/A | Yes | NSF | NSF |
| MKS-1704-Lc | < MDL | < MDL | Yes | Hepatitis (Lymphoplasmacytic, Histiocytic, Multifocal, Moderate); Hepatocellular Necrosis/Loss; Dysfunction possible | NSF |
| MKS-1706-Lc | < MDL | < MDL | Yes | Hepatitis (Necrotizing, Mixed inflammatory, Active, Multifocal, Mild-Moderate); No causative agents observed | NSF |
| MKS-1708-Lc | N/A | N/A | Yes | NSF | NSF |
| MKS-1711-Lc | N/A | N/A | Yes | NSF | NSF |
| MKS-1712-Lc | < MDL | N/A | Yes | NSF | NSF |
| MKS-1716-Lc | N/A | N/A | Yes | NSF | NSF |
| MKS-1718-Lc | < MDL | < MDL | Yes | Hyperplasia (Nodular, Multifocal, Moderate) | NSF |
| MKS-1719-Lc | < MDL | N/A | Yes | Fracture | NSF |

| | | | | | |
|---|---|---|---|---|---|
| MKS-1720-Lc | N/A | N/A | Yes | Hyperplasia (Nodular, Focal); Lipidosis (Multifocal, Suspect); Fracture | NSF |
| MKS-1722-Lc | < MDL | N/A | Yes | NSF | NSF |
| MKS-1724-Lc | < MDL | < MDL | Yes | Hepatitis (Portal, Lymphoplasmacytic, Focal) | Medullary Dilation and Mineralization (Focal, Mild) |
| MKS-1725-Lc | < MDL | N/A | Yes | Fracture | NSF |
| MKS-1727-Lc | < MDL | N/A | Yes | NSF | NSF |
| MKS-1802-Lc | N/A | N/A | Yes | Fracture | NSF |
| MKS-1811-Lc | < MDL | N/A | No | N/A | N/A |
| MKS-1813-Lc | < MDL | N/A | Yes | N/A | N/A |
| MKS-1815-Lc | < MDL | N/A | Yes | N/A | N/A |
| MKS-1817-Lc | < MDL | N/A | No | N/A | N/A |
| MKS-1818-Lc | < MDL | N/A | No | N/A | N/A |
| MKS-1819-Lc | < MDL | N/A | No | N/A | N/A |
| MKS-1901-Lc | < MDL | N/A | No | N/A | N/A |
| MKS-1902-Lc | < MDL | N/A | No | N/A | N/A |
| MKS-1910-Lc | < MDL | N/A | No | NSF | NSF |
| MKS-1921-Lc | < MDL | N/A | No | N/A | N/A |
| MKS-2007-Lc | < MDL | N/A | No | N/A | N/A |
| MKS-2009-Lc | < MDL | N/A | No | N/A | N/A |
| MKS-2011-Lc | < MDL | N/A | No | NSF | NSF |

| | | | | | |
|---|---|---|---|---|---|
| MKS-2012-Lc | < MDL | N/A | No | N/A | N/A |
| MKS-2015-Lc | < MDL | N/A | No | NSF | NSF |
| MKS-2102-Lc | < MDL | N/A | No | N/A | N/A |
| MKS-2110-Lc | < MDL | N/A | No | Fracture | NSF |
| MKS-2116-Lc | < MDL | N/A | No | NSF | NSF |
| MKS-2117-Lc | < MDL | N/A | No | NSF | Nephritis (Interstitial, Lymphoplasmacytic, Multifocal, Mild) |
| MKS-2201-Lc | < MDL | N/A | No | NSF | NSF |
| MKS-2206-Lc | < MDL | N/A | No | Hepatitis (Granulomatous, Multifocal, Mild-Moderate); Dysfunction possible | NSF |
| MKS-2212-Lc | < MDL | N/A | No | N/A | N/A |
| MKS-2215-Lc | < MDL | N/A | No | N/A | N/A |

Table S3. Trace element levels (µg/g dry weight [dw]) in the liver of IRL river otters ($n$ = 22) measured by inductively coupled plasma mass spectrometry (ICP-MS) (2016-2018).

| Sender ID | Dry fraction | As | Cd | Pb | Hg | Co | Tl | Se | Fe | Cu | Zn | Mo | Mn |
|---|---|---|---|---|---|---|---|---|---|---|---|---|---|
| MKS-1603-Lc | 0.336 | 0 | 0.16 | 0 | 1.16 | 0.04 | 0 | 1.99 | 326.77 | 36.06 | 89.13 | 2.23 | 9.48 |
| MKS-1604-Lc | 0.309 | 0 | 0.09 | 0 | 1.7 | 0 | 0 | 1.97 | 657.76 | 34.99 | 82.73 | 2.37 | 8.93 |
| MKS-1605-Lc | 0.334 | 0 | 0 | 0.11 | 1.47 | 0.09 | 0 | 2.02 | 689.36 | 30.58 | 90.02 | 2.35 | 12.31 |
| MKS-1607-Lc | 0.307 | 0 | 0 | 0.41 | 1.12 | 0.04 | 0 | 2.31 | 943.48 | 36 | 81.17 | 3.01 | 12.37 |
| MKS-1610-Lc | 0.329 | 0 | 0.33 | 0 | 3.56 | 0 | 0 | 2.57 | 136.1 | 23.99 | 106.47 | 4.15 | 21.26 |
| MKS-1702-Lc | 0.31 | 0 | 0 | 0.11 | 0 | 0.04 | 0 | 1.9 | 296.44 | 149.57 | 100.03 | 2.19 | 12.85 |
| MKS-1704-Lc | 0.345 | 0 | 0.19 | 0 | 3.56 | 0.04 | 0 | 1.87 | 145.64 | 34.36 | 79.62 | 2.51 | 13.04 |
| MKS-1706-Lc | 0.306 | 0 | 0.27 | 0.11 | 1.31 | 0 | 0 | 2.69 | 204.46 | 39.35 | 88.43 | 2.63 | 12.95 |
| MKS-1708-Lc | 0.325 | 0 | 0.44 | 0.2 | 8.38 | 0.04 | 0 | 2.3 | 773.4 | 40.25 | 98.64 | 3.15 | 11.2 |
| MKS-1711-Lc | 0.295 | 0.29 | 0 | 0.54 | 1.75 | 0 | 0 | 2.64 | 407.05 | 54.83 | 91.96 | 1.68 | 11.79 |
| MKS-1712-Lc | 0.326 | 0 | 0.68 | 0 | 3.57 | 0.05 | 0 | 2.6 | 473.18 | 21.81 | 91.74 | 3.48 | 8.55 |
| MKS-1716-Lc | 0.305 | 0.31 | 0.09 | 0.28 | 0.52 | 0.04 | 0 | 2.67 | 203.79 | 51.32 | 171.06 | 3.43 | 15.35 |
| MKS-1718-Lc | 0.314 | 0 | 0 | 0.2 | 0.71 | 0.04 | 0 | 2.96 | 1047.74 | 15.9 | 74.66 | 2.29 | 6.55 |
| MKS-1719-Lc | 0.339 | 0 | 0.09 | 0 | 2.24 | 0 | 0 | 2.16 | 234.89 | 42.93 | 70.84 | 2.69 | 9.75 |
| MKS-1720-Lc | 0.322 | 0 | 0.26 | 0.28 | 1.09 | 0.06 | 0 | 2.34 | 971.53 | 15.96 | 83.13 | 3.29 | 9.08 |
| MKS-1722-Lc | 0.321 | 0 | 0.28 | 0.22 | 2.23 | 0 | 0 | 3.21 | 1222.46 | 39.2 | 96.57 | 2.91 | 9.17 |

| | | | | | | | | | | | | | |
|---|---|---|---|---|---|---|---|---|---|---|---|---|---|
| MKS-1724-Lc | 0.319 | 0 | 0.17 | 0 | 1.58 | 0.06 | 0 | 1.51 | 638.03 | 18.4 | 69.38 | 2.68 | 7.39 |
| MKS-1725-Lc | 0.329 | 0 | 0.09 | 0 | 3.17 | 0 | 0 | 2.71 | 300.66 | 79.03 | 104.78 | 2.06 | 9.7 |
| MKS-1727-Lc | 0.292 | 0 | 0 | 0.09 | 1.19 | 0 | 0 | 1.97 | 566.88 | 21.05 | 82.23 | 1.91 | 7.2 |
| MKS-1802-Lc | 0.3 | 0 | 0.12 | 0 | 3.1 | 0.04 | 0 | 2.51 | 343.45 | 46.15 | 109.78 | 3.31 | 13.71 |
| MKS-1813-Lc | 0.308 | 0 | 0.5 | 0.19 | 2.55 | 0.06 | 0 | 2.57 | 859.72 | 43.04 | 95.44 | 3.04 | 9.05 |
| MKS-1815-Lc | 0.325 | 0.09 | 0.09 | 0.17 | 2.43 | 0 | 0 | 2.25 | 1565.26 | 54.8 | 92.55 | 2.93 | 5.26 |

Table S4. Trace element levels (μg/g dw) in the kidney of IRL river otters (*n* = 22) measured by ICP-MS (2016-2018).

| Sender ID | Dry fraction | As | Cd | Pb | Hg | Co | Tl | Se | Fe | Cu | Zn | Mo | Mn |
|---|---|---|---|---|---|---|---|---|---|---|---|---|---|
| MKS-1603-Lc | 0.384 | 0 | 0.7 | 0 | 0.78 | 0.03 | 0 | 3.69 | 196.08 | 7.74 | 48.51 | 0.57 | 1.46 |
| MKS-1604-Lc | 0.274 | 0 | 0.59 | 0 | 1.49 | 0 | 0 | 4.5 | 171.65 | 10.63 | 71.32 | 0.78 | 2.15 |
| MKS-1605-Lc | 0.269 | 0 | 0.22 | 0.1 | 1.08 | 0 | 0 | 3.64 | 269.18 | 10.96 | 70.15 | 0.74 | 2.19 |
| MKS-1607-Lc | 0.241 | 0 | 0.26 | 0.4 | 0.92 | 0 | 0 | 4.14 | 271.9 | 11.08 | 72.31 | 0.77 | 1.85 |
| MKS-1610-Lc | 0.262 | 0 | 1.3 | 0 | 4.36 | 0 | 0 | 3.74 | 229.75 | 13.15 | 79.08 | 0.81 | 3.38 |
| MKS-1702-Lc | 0.247 | 0 | 0 | 0 | 0 | 0 | 0 | 4.65 | 290.39 | 14 | 77.94 | 0.69 | 3 |
| MKS-1704-Lc | 0.266 | 0 | 1.21 | 0 | 4.3 | 0.05 | 0 | 4.4 | 188.73 | 14.1 | 73.25 | 0.86 | 3.39 |
| MKS-1706-Lc | 0.244 | 0 | 1.01 | 0.1 | 1.16 | 0 | 0 | 4.56 | 255.61 | 19.59 | 74.71 | 0.7 | 2.71 |
| MKS-1708-Lc | 0.258 | 0 | 1.76 | 0.15 | 6.57 | 0.06 | 0 | 4.39 | 251.76 | 13.45 | 73.72 | 0.76 | 2.96 |
| MKS-1711-Lc | 0.244 | 0.52 | 0 | 0.38 | 1.5 | 0 | 0 | 4.75 | 398.51 | 9.99 | 69 | 0.48 | 1.88 |
| MKS-1712-Lc | 0.257 | 0 | 3.51 | 0 | 3.78 | 0 | 0 | 5 | 263.25 | 12.8 | 74.49 | 0.87 | 1.71 |
| MKS-1716-Lc | 0.307 | 0.39 | 0.34 | 0 | 0.44 | 0 | 0 | 2.86 | 268.44 | 10.18 | 105.43 | 0.68 | 2.12 |
| MKS-1718-Lc | 0.473 | 0 | 0.12 | 0.1 | 0.5 | 0 | 0 | 2.44 | 237.88 | 6.33 | 35.49 | 0.3 | 0.84 |
| MKS-1719-Lc | 0.236 | 0 | 0.54 | 0 | 2.56 | 0 | 0 | 4.51 | 256.69 | 13.55 | 70.92 | 0.7 | 2.52 |
| MKS-1720-Lc | 0.261 | 0 | 0.59 | 0.2 | 1.43 | 0 | 0 | 4.29 | 514.88 | 11.82 | 71.34 | 0.75 | 3.46 |
| MKS-1722-Lc | 0.268 | 0 | 1.42 | 0.12 | 2.03 | 0 | 0 | 4.81 | 476.81 | 14.16 | 74.74 | 0.79 | 3.2 |
| MKS-1724-Lc | 0.271 | 0 | 0.74 | 0 | 2.04 | 0 | 0 | 3.75 | 497.59 | 12.91 | 66.71 | 0.74 | 2.29 |

| | | | | | | | | | | | | | |
|---|---|---|---|---|---|---|---|---|---|---|---|---|---|
| MKS-1725-Lc | 0.257 | 0 | 0.11 | 0 | 2.94 | 0 | 0 | 3.02 | 605.36 | 52.03 | 91.77 | 2.22 | 9.81 |
| MKS-1727-Lc | 0.288 | 0 | 0.2 | 0.17 | 1.25 | 0 | 0 | 3.49 | 509.75 | 14.77 | 68.68 | 0.7 | 2.71 |
| MKS-1802-Lc | 0.271 | 0 | 0.36 | 0 | 2.47 | 0 | 0 | 4.13 | 413.89 | 15.77 | 72.46 | 0.81 | 3.34 |
| MKS-1813-Lc | 0.253 | 0 | 2.73 | 0.13 | 2.33 | 0 | 0 | 4.84 | 262.78 | 13.51 | 77.93 | 0.85 | 2.62 |
| MKS-1815-Lc | 0.245 | 0.14 | 0.27 | 0 | 1.85 | 0 | 0 | 4.43 | 754.12 | 13.68 | 65.72 | 0.87 | 1.67 |

Table S5. The severity of microscopic inflammation in IRL river otters evaluated for MC and/or trace element bioaccumulation (2016-2022) with non-traumatic liver and/or kidney histopathologic anomalies (*n* = 7). NSF = no significant histopathological findings, + = mild inflammation, ++ = moderate inflammation, +++ = severe inflammation

| **Sender ID** | **Liver histopathology** | **Inflammation severity** | **Kidney histopathology** | **Inflammation severity** |
|---|---|---|---|---|
| MKS-1704-Lc | Hepatitis (lymphoplasmacytic, histiocytic, multifocal), Hepatocellular necrosis/loss | ++ | NSF | |
| MKS-1706-Lc | Hepatitis (necrotizing, mixed inflammatory, active, multifocal) | ++ | NSF | |
| MKS-1718-Lc | Hyperplasia (nodular, multifocal, moderate) | none | NSF | |
| MKS-1720-Lc | Hyperplasia (nodular, focal), Lipidosis (multifocal, suspect) | none | NSF | |
| MKS-1724-Lc | Hepatitis (portal, lymphoplasmacytic, focal) | + | Medullary dilation and mineralization (focal, mild) | none |
| MKS-2117-Lc | NSF | | Nephritis (interstitial, lymphoplasmacytic, multifocal) | + |
| MKS-2206-Lc | Hepatitis (granulomatous, multifocal) | ++ | NSF | |

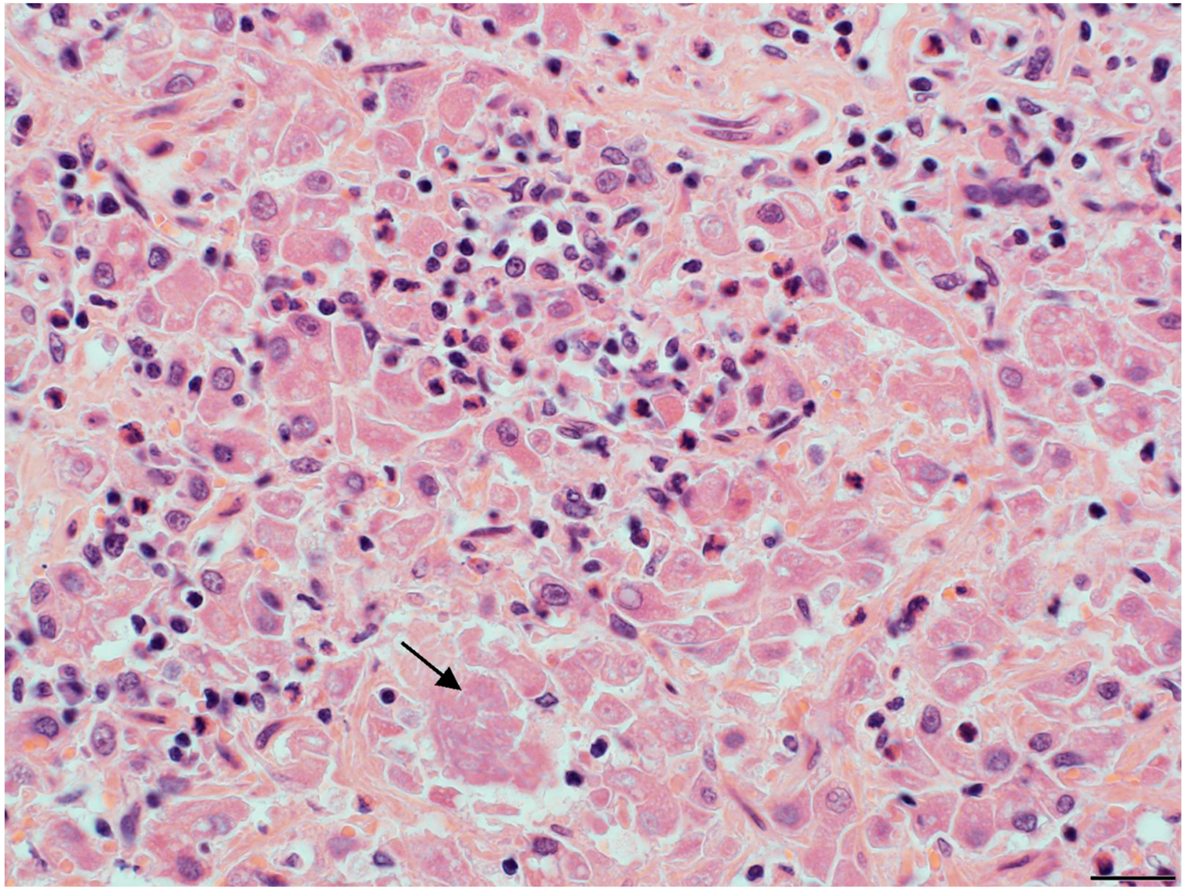

Figure S1. Moderate, multifocal hepatitis (lymphoplasmacytic and histiocytic) and hepatocellular necrosis/loss (arrow) in an IRL river otter (MKS-1704-Lc); H & E, 300 dpi, 40x, scale bar = 20 µm. Image credit/Histologic interpretation: Dr. David Rotstein.

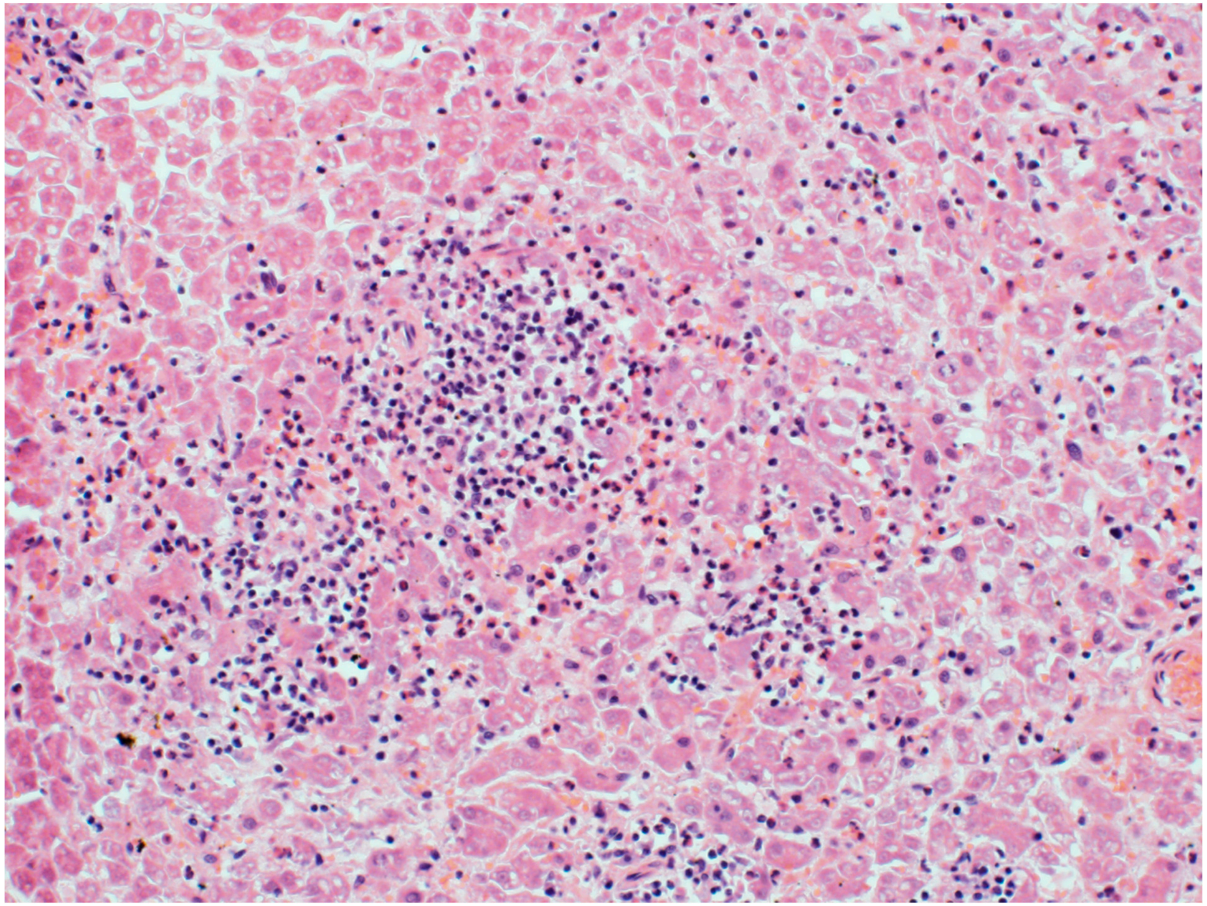

Figure S2. Mild-moderate, multifocal, active hepatitis (necrotizing and mixed inflammatory) in an IRL river otter (MKS-1706-Lc); H & E, 300 dpi, 20x.

Image credit/Histologic interpretation: Dr. David Rotstein.

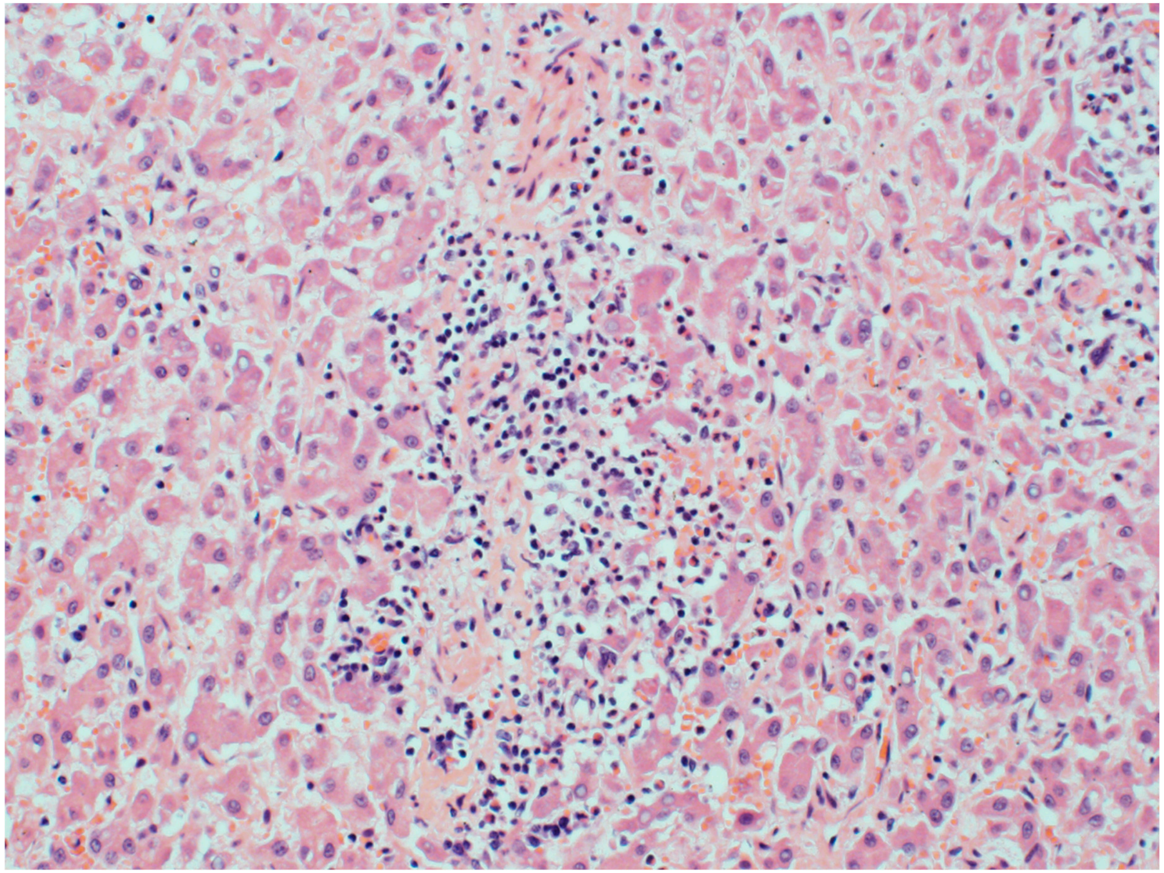

Figure S3. Focal portal hepatitis (lymphoplasmacytic) in an IRL river otter (MKS-1724-Lc); H & E, 300 dpi, 20x.

Image credit/Histologic interpretation: Dr. David Rotstein.

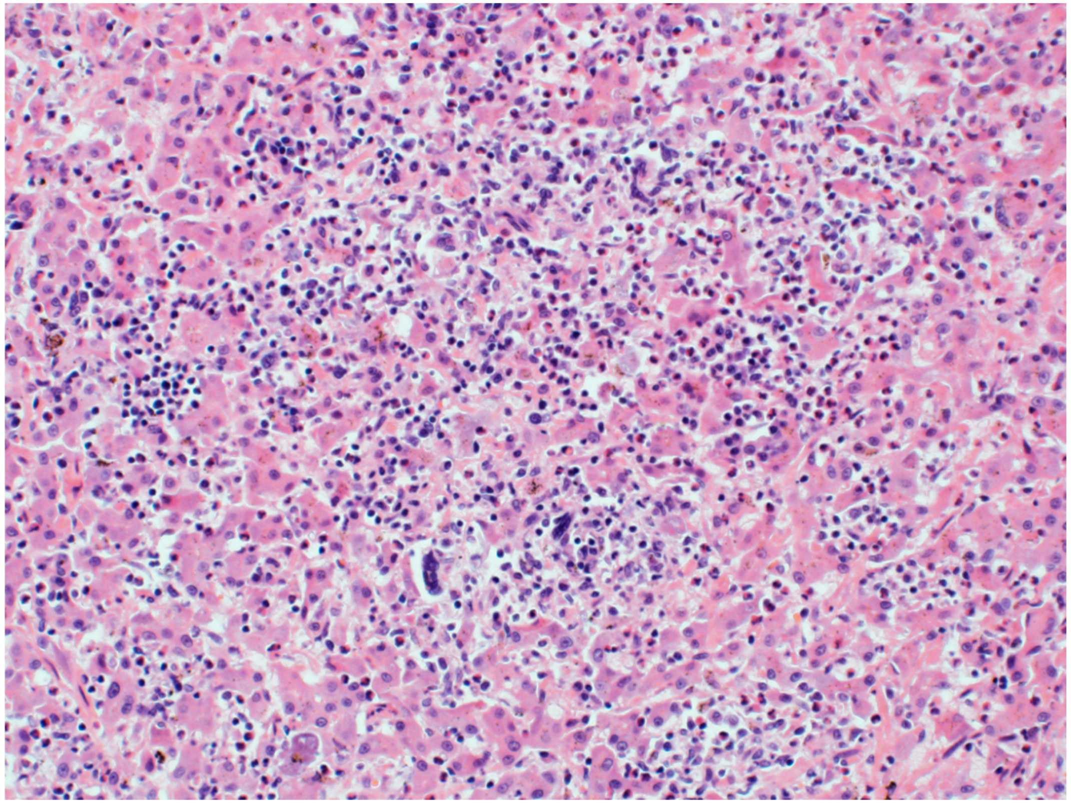

Figure S4. Mild-moderate multifocal hepatitis (granulomatous) in an IRL river otter (MKS-2206-Lc); H & E, 300 dpi, 20x.

Image credit/Histologic interpretation: Dr. David Rotstein.

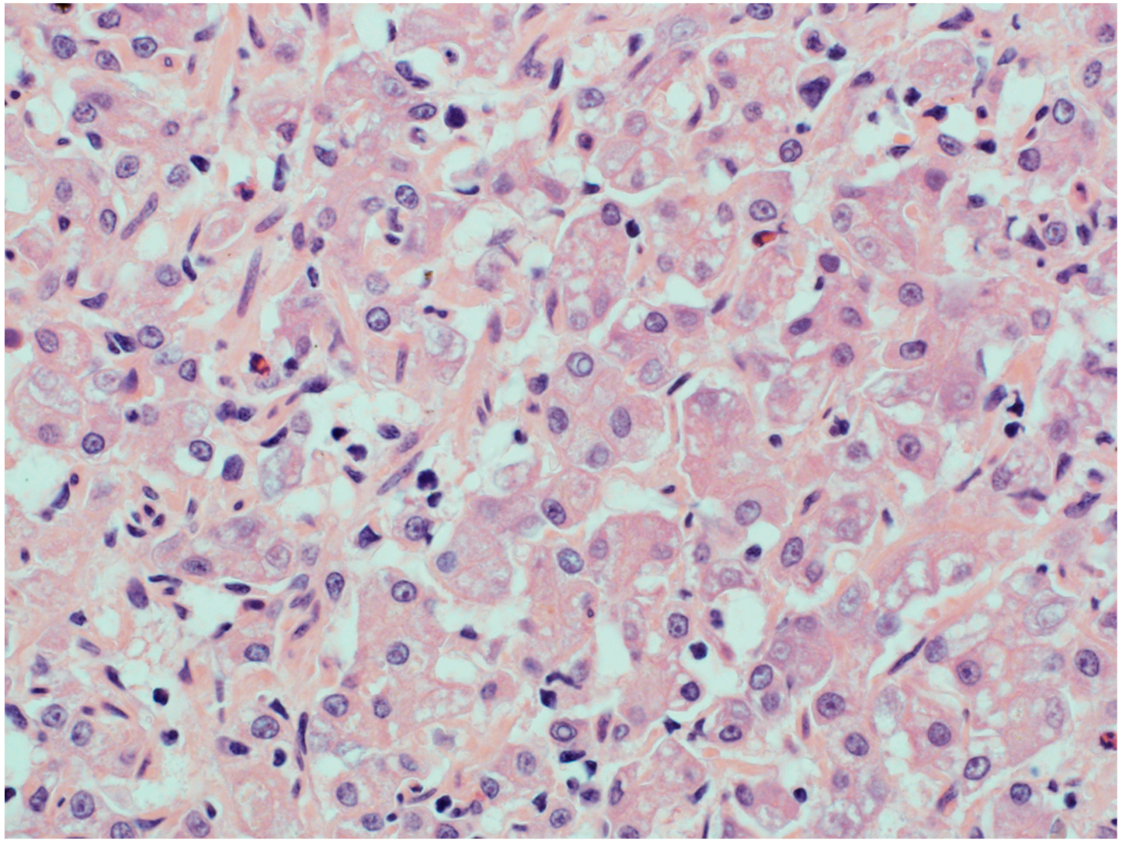

Figure S5. Multifocal hepatic lipidosis (suspect) and focal nodular hyperplasia in an IRL river otter (MKS-1720-Lc); H & E, 300 dpi, 40x.

Image credit/Histologic interpretation: Dr. David Rotstein.

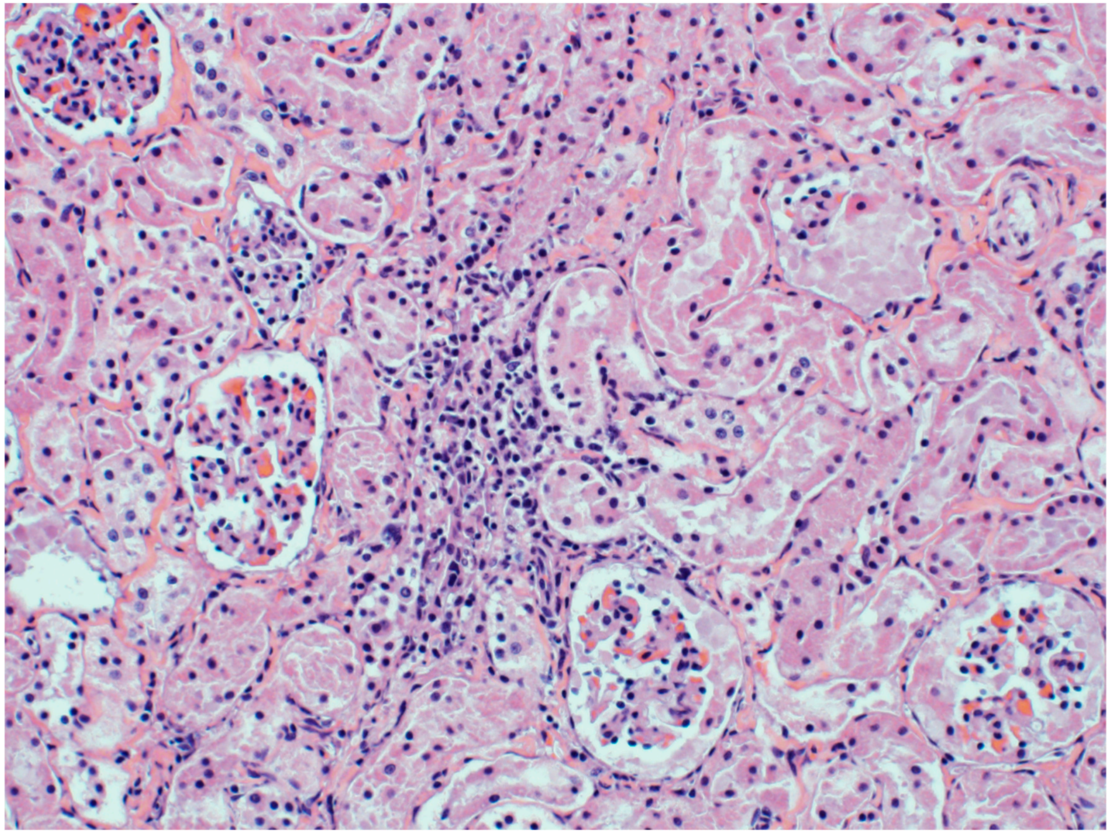

Figure S6. Mild, multifocal interstitial nephritis (lymphoplasmacytic) in an IRL river otter (MKS-2117-Lc); H & E, 300 dpi, 20x.

Image credit/Histologic interpretation: Dr. David Rotstein.

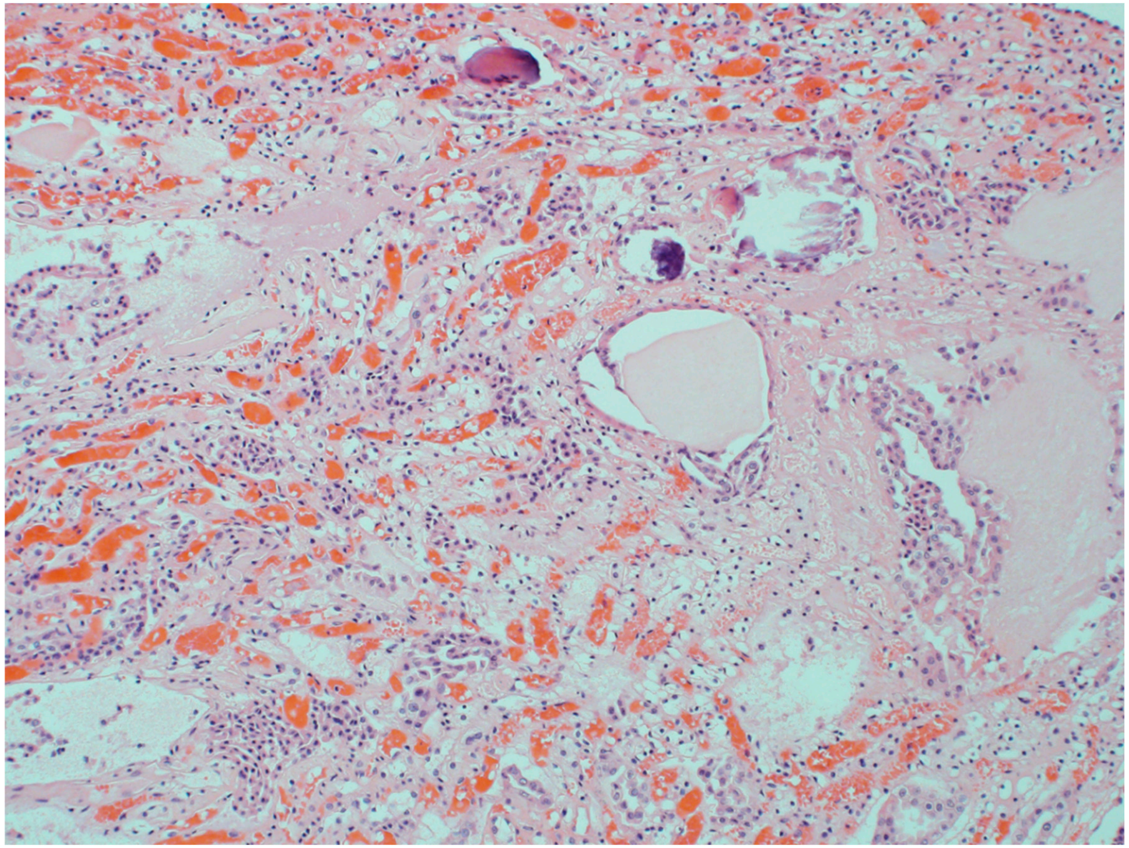

Figure S7. Mild, focal medullary dilation and mineralization in the kidney of an IRL river otter (MKS-1724-Lc); H & E, 300 dpi, 10x.

Image credit/Histologic interpretation: Dr. David Rotstein.
